# Binding Polymorphism in the DNA Bound State of the Pdx1 Homeodomain

**DOI:** 10.1371/journal.pcbi.1003160

**Published:** 2013-08-08

**Authors:** Volodymyr Babin, Dongli Wang, Robert B. Rose, Celeste Sagui

**Affiliations:** 1 Center for High Performance Simulations (CHiPS) and Department of Physics, North Carolina State University, Raleigh, North Carolina, United States of America; 2 Department of Chemistry and Biochemistry, UC San Diego, La Jolla, California, United States of America; 3 Department of Molecular and Structural Biochemistry, North Carolina State University, Raleigh, North Carolina, United States of America; University of Maryland, Baltimore, United States of America

## Abstract

The subtle effects of DNA-protein recognition are illustrated in the homeodomain fold. This is one of several small DNA binding motifs that, in spite of limited DNA binding specificity, adopts crucial, specific roles when incorporated in a transcription factor. The homeodomain is composed of a 3-helix domain and a mobile N-terminal arm. Helix 3 (the recognition helix) interacts with the DNA bases through the major groove, while the N-terminal arm becomes ordered upon binding a specific sequence through the minor groove. Although many structural studies have characterized the DNA binding properties of homeodomains, the factors behind the binding specificity are still difficult to elucidate. A crystal structure of the Pdx1 homeodomain bound to DNA (PDB 2H1K) obtained previously in our lab shows two complexes with differences in the conformation of the N-terminal arm, major groove contacts, and backbone contacts, raising new questions about the DNA recognition process by homeodomains. Here, we carry out fully atomistic Molecular Dynamics simulations both in crystal and aqueous environments in order to elucidate the nature of the difference in binding contacts. The crystal simulations reproduce the X-ray experimental structures well. In the absence of crystal packing constraints, the differences between the two complexes increase during the solution simulations. Thus, the conformational differences are not an artifact of crystal packing. In solution, the homeodomain with a disordered N-terminal arm repositions to a partially specific orientation. Both the crystal and aqueous simulations support the existence of different stable binding conformers identified in the original crystallographic data with different degrees of specificity. We propose that protein-protein and protein-DNA interactions favor a subset of the possible conformations. This flexibility in DNA binding may facilitate multiple functions for the same transcription factor.

## Author Summary

All organisms require the capability to control gene expression. In eukaryotes, transcription factors play an important role in gene regulation by recognizing specific DNA control regions associated with each gene. The DNA binding domains of transcription factors belong to evolutionarily conserved families with different protein folds. An example is the homeodomain family. Although this DNA binding domain has been studied for a long time, the properties that determine DNA binding specificity are still not clear. We previously showed in a crystal structure that the homeodomain of a transcription factor Pdx1 (Pancreatic and duodenal homeobox 1) binds DNA in 2 different conformations. In this paper, we used Molecular Dynamics simulations to show that both of these conformations are stable in solution. This is surprising since it is often assumed that proteins recognize DNA by finding a single lowest energy state. This study shows that transcription factors may bind DNA in an ensemble of conformations. This scenario may facilitate their finding the correct binding site among the 3 billion basepairs of DNA in the human genome. It may also provide flexibility in the DNA sequence that homeodomains can recognize to promote gene transcription.

## Introduction

Specific DNA binding plays a key role in the protein-DNA recognition process necessary for the regulation of gene expression. Binding determinants are complex, including direct amino acid-base contacts, indirect water-mediated contacts, and local geometry of the DNA sequence [1]. Although it is possible to identify certain trends in the recognition process, such as the formation of point contacts between certain base pairs and certain amino acids, at present there are no unequivocal correspondences between bases and amino acids. Complicating matters, many regions of transcription factors are disordered in solution and fold only upon binding to their specific targets [2]. The current study proposes an additional level of complexity, suggesting that the bound state may also consist of an ensemble of stable conformations instead of a single low energy conformation.

The homeodomain fold provides an interesting example of the subtle effects of DNA-protein recognition. The homeodomain is one of several small DNA binding motifs with limited DNA binding specificity, yet incorporated in an estimated 235 transcription factors it adopts specific and essential developmental roles [3], [4], [5]. The homeodomain is composed of a 3-helix domain and a mobile N-terminal arm. Helix 2 and 3 form a helix-turn-helix type motif that is ordered in solution. Helix 3, also known as the recognition helix, interacts with the DNA bases through the major groove. The N-terminal arm, on the other hand, becomes ordered upon binding a specific DNA sequence through the minor groove [6], [7], [8].

Homeodomain factors participate in a wide range of functions. The current study focuses on Pdx1 (Pancreatic and duodenal homeobox 1), a ParaHox transcription factor evolutionarily related to the Hox subfamily of homeodomains. Hox homeodomains regulate body plan development from *Drosophila* to humans [9], [10], [11]. Genome-wide binding studies of Hoxa2 and Pdx1 indicate that they may regulate thousands of genes [12], [13]. Pdx1 regulates differentiation of the duodenum and stomach, and is a master regulator of pancreas development [14], [15], [16], [17]. In the mature pancreas Pdx1 is expressed in beta- and delta-cells that secrete the endocrine hormones insulin and somatostatin, respectively. Mutations in Pdx1 cause a form of familial diabetes, maturity-onset diabetes of the young type 4 (MODY-4) [18], [19].

How homeodomain factors achieve functional diversity as well as exquisite specificity remains a subject of debate. Many studies have correlated DNA binding affinity of homeodomain factors with *in vivo* activity [20], [21], [22], [23], [24]. DNA binding affinity of Pdx1 monomers accounts for differences in transcriptional activity, at least in cell culture [23]. Hox binding sites include the TAAT core consensus sequence, cooperative binding sites with the TALE homeodomain factors, and sites with no recognizable binding motif [12], [13]. Interactions with the TALE factors PBC and Meis alters DNA binding specificity of the Hox homeodomains [25], [26], [27], [28]. Pdx1 cooperates with Pbx1 and Prep1 on the somatostatin promoter, [29], [30], Pbx1 and Mrg1 in pancreatic acinar cells [31], and the basic-helix-loop-helix factor E47/NeuroD on the insulin promoter [32], [33], [34]. Disordered sequences outside of the homeodomain can influence DNA binding specificity suggesting an auto-inhibitory mechanism [35]. Additionally phosphorylation or sumoylation, important for nuclear localization, may affect activity [36], [37], [38]. Diversity is also achieved through ‘activity regulation’, by recruiting different coactivators and corepressors to non-conserved regions outside of the homeodomain [22], [39].

**Figure 1:**
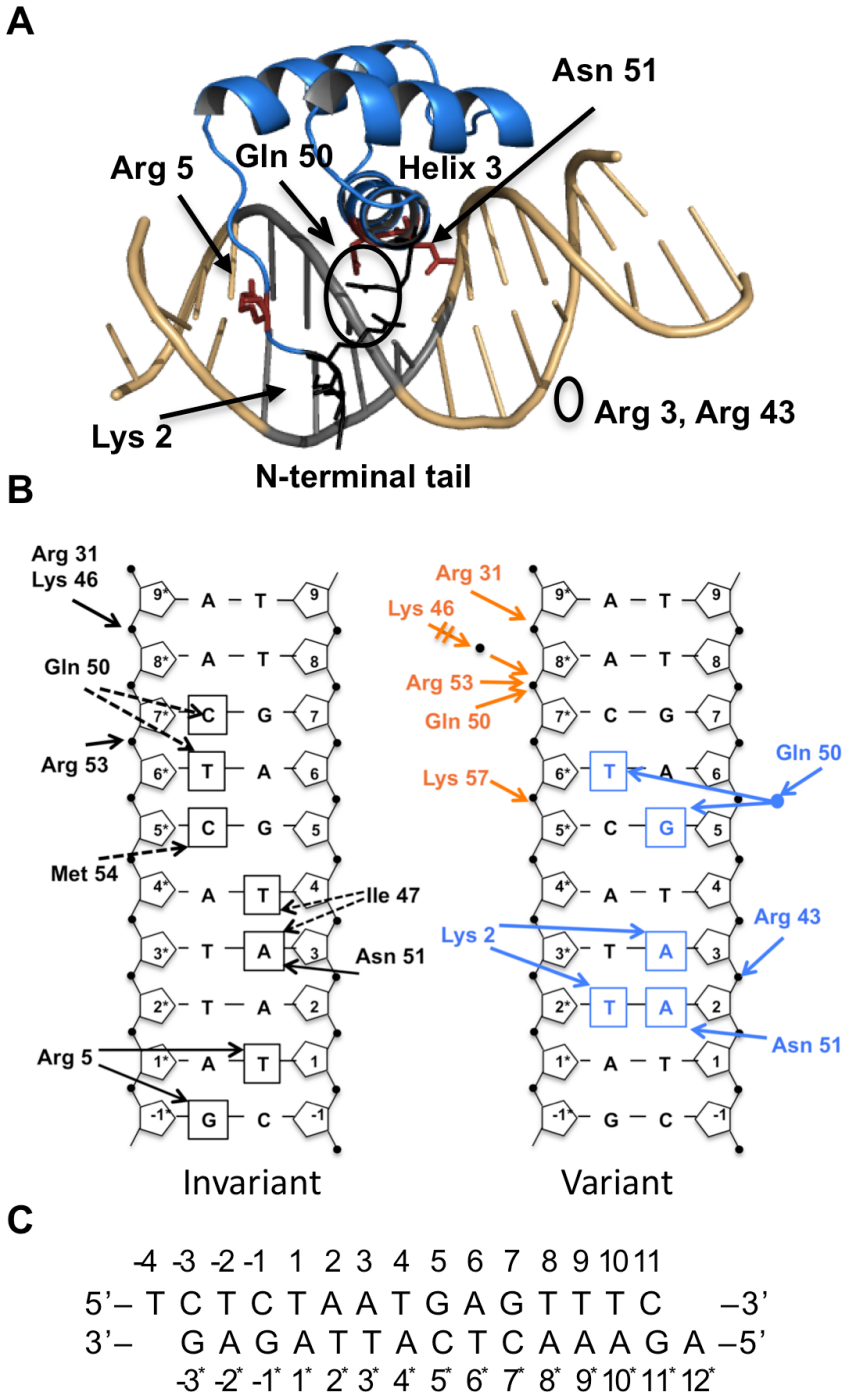
Pdx1 homeodomain/DNA interactions from the crystal structure. A) Structure of the Pdx1 homeodomain/DNA complex. Pdx1 (blue ribbon) binds the TAAT core DNA sequence (grey). The N-terminal tail binds in the minor groove, and the recognition helix, helix 3, binds in the major groove. Key residues contacting the DNA are shown as stick figures (red): Arg 5 in the minor groove, and Asn 51 in the major groove. Gln 50 contacts the phosphate backbone or the DNA bases through a water-mediated contact. Arg 3 and Arg 43 (black line representation, circled) help stabilize the N-terminal arm, and Lys 2, in the minor groove when helix 3 is properly positioned in the major groove. B) Hydrogen bond contacts with the DNA differ between Conformation A and B in the Pdx1 homeodomain/DNA crystal structure (PDB 2H1K) (www.rcsb.org) [56]. In both conformations (left) Arg 5 contacts Thy 1 and Gua −1* through the minor grove, and Asn 51 contacts Ade 3 through the major grove. The difference in the DNA contacts between Conformations A (orange) and B (blue) is shown on the right. Conformation B makes additional base contacts by Asn 51, by Lys 2 from the ordered N-terminal arm, and a water-mediated contact by Gln 50. Conformation A forms additional phosphate contacts. Arrows represent hydrogen bonds. C) DNA sequence and numbering in the crystal structure. The TAAT core sequence is in bold.

Many structural studies have characterized the DNA binding properties of homeodomains. All Hox factors contact DNA through similar residues (Figure 1A). Residues Ile 47, Gln 50, Asn 51 and Met 54 from the recognition helix insert in the major groove contacting DNA bases directly or through water bridges [40]. Position 50 is particularly important for specificity for some homeodomains, for example Lys 50 in Bicoid [41], but less so for Hox factors as demonstrated by a Gln 50 to Ala mutation [42]. The conservation of the major groove residues suggests they are insufficient to distinguish binding specificity among Hox factors.

The N-terminal arm sequence is less well conserved than the recognition helix, but typically includes positively charged Lys or Arg residues [43], [44]. The arm sequence contributes to DNA binding specificity as demonstrated by chimeric homeodomains with swapped N-terminal residues [24], [45], [46], [47]. Even so the N-terminal arm is often disordered in crystal structures of homeodomain monomers bound to DNA. Coarse-grained Molecular Dynamics simulations indicate that the disordered N-terminal arm facilitates searching the DNA for binding sites through electrostatic attraction by a sliding mechanism or transferring between DNA strands by a “fly catching” mechanism [44], [48], [49], [50].

Recently a crystal structure of the Pdx1 homeodomain/DNA complex was obtained in our lab with a consensus DNA binding sequence C_−1_T_1_A_2_A_3_T_4_G_5_A_6_G_7_ [51]. The structure contained two complexes with differences in the conformation of the N-terminal arm, major groove contacts, and backbone contacts, raising new questions about the DNA recognition process by homeodomains (Figure 1 B,C) [51]. At the time we attributed the differences in the two conformations to differences in DNA bending as a result of crystal packing [51]. We proposed an induced fit model in which DNA contacts by residues from helix 3 in the major groove of one conformation stabilized the N-terminal arm in the minor groove.

In this work we apply classical Molecular Dynamics (MD) with a fully atomistic representation of the complex and solvent to simulate both the crystal and solution behavior of both conformations of the Pdx1 homeodomain/DNA complex [51]. In the last decade MD simulations have become an invaluable tool to complement structural information obtained experimentally [52], [53], [54], [55]. MD simulations of the Pdx1/DNA complexes show that differences in DNA contacts persist between the two conformations even in solution due to distinct positioning of the homeodomain relative to the DNA. Conformation A represents a less specific complex than Conformation B. The simulations suggest that one source of diversity of homeodomain function derives from different bound states with different degrees of DNA specificity. The existence of these “isomeric” bound conformations has not been reported before. We propose that multiple bound isomers are an important feature of the homeodomain/DNA binding processes, adding another layer of complexity to what is known about binding specificity.

## Materials and Methods

### Simulation details

Simulations were carried out for: (i) the crystal unit cell; (ii) aqueous solution; and (iii) the DNA and Pdx1 molecules separately. Initial geometries for the simulations were derived from both Pdx1/DNA complexes in the asymmetric unit of the crystal structure (pdbid 2H1K) (www.rcsb.org) [51], [56]. Residues missing in the crystal structure (model A: residues 1–3, 60–61; model B: residues 58–61) were placed in low energy conformations by superimposing short pre-equilibrated peptide fragments onto the experimental structure. For the solution simulations all waters from the crystal structure were removed and replaced with solvent water molecules surrounding the protein/DNA complex.

For the crystal simulation the unit cell of the crystal structure was generated from the asymmetric unit by applying the P212121 symmetry operators. Non-crystallographic water molecules were added by sampling them from a box of water equilibrated at constant pressure and temperature conditions and placed “on top” of the unit cell. Specifically, molecules were picked at random from the water box and “copied” into the unit cell provided no sterically forbidden configuration results. The number of the water molecules was varied until the system's density remained unchanged in trial MD runs under normal conditions. The density settled at 1.25 g/cm3 with 6901 non-crystallographic waters.

Simulations were performed using the AMBER 10 package along with some “in house” codes [57]. The Pdx1/DNA complex was modeled using the ff99SB [58] force field for protein, parmbsc0 [59] for DNA, TIP3P [60] for water molecules and the AMBER stock 1999 version of the Cornell force field [61] for Na^+^ and Cl^−^ ions. Missing hydrogen atoms were added with the LEaP module of AMBER 10; histidine residues were assumed to be neutral with a proton at the ε2 position. For the solution simulations, at least a 15 Å thick layer of TIP3P water was added around the solute with the LEaP module, and the system was neutralized using either Na^+^ (Pdx1/DNA complex) or Cl^−^ (protein alone) ions. The structures were thoroughly equilibrated with the SANDER module of AMBER 10 before the production (data gathering) step. During the initial equilibration the heavy DNA and protein atoms were restrained to their initial positions by a harmonic potential. In every case we first performed a few conjugate gradient minimization steps to relax the hydrogens, followed by a 1 nanosecond long constant pressure run at ambient conditions (T = 298 K, P = 1 atm). The last frame of the restrained NPT runs were the starting point of the *unrestrained* NPT production runs reported in this paper.

The production simulations were carried out using the PMEMD module of AMBER 10. The electrostatic interactions were evaluated by the PME method [62], [63] using a 9 Å cutoff for the short-range terms. The same cutoff was used for the van der Waals terms with a continuous correction for the long-range terms. The lengths of all bonds that involve hydrogen atoms were fixed via the SHAKE algorithm with the tolerance set to 10^−6^ Å. Langevin dynamics [64] with collision frequency γ = 1 ps^−1^ (ps = picosecond) was used to maintain the temperature at 298 K with a different random number generator seed set for every run. The Berendsen algorithm [65] with relaxation time τ_P_ = 1 ps was used to maintain the pressure at 1 atm. The total time for each simulation was 50 ns with a time step of 2 femtoseconds and coordinates saved for analysis every 10 ps (5000 steps).

### Analysis

The PTRAJ module of AMBER 10 was used for basic analysis (centering and imaging of the trajectories, computations of RMS deviations, etc.), 3DNA (v. 1.5) [66] for the calculation of DNA structural parameters, and simple in-house programs for the identification and counting of the intermolecular contacts. The latter were defined as follows [67]: a hydrogen bond was assumed if the distance between the donor hydrogen and the accepter oxygen or nitrogen was 2.8 Å or less, and the angle formed by the donor, hydrogen and acceptor atoms exceeded 145°; a hydrophobic contact was defined as a pair of sulfur/carbon atoms separated by less than 4.5 Å; a water contact was identified if the oxygen of a water molecule was within 3 Å of a nitrogen or an oxygen atom. A simultaneous water contact from two different macromolecules to the same water molecule is referred to as “water bridge”.

Figures of protein structures were generated with Pymol [68] and labeled in Powerpoint (Microsoft Office). The molecular graphics image of the unit cell was produced using the UCSF program Chimera [69]. The figures displaying distances through the simulation are displayed as a running average of 100 ps (10 trajectory frames).

## Results

### Conformation-specific DNA contacts in the crystal structure

The two conformations of the Pdx1/DNA complex in the crystal structure contained invariant contacts found in both conformations A and B, and variable contacts specific to each conformation (Figure 1B) [51]. Two residues formed direct hydrogen bonds with DNA bases in both conformations: Asn 51 with Ade 3 (CTAA_3_T) in the major groove, and Arg 5 with Thy 1 (C_−1_ T_1_AAT) and Gua −1* (opposite Cyt −1) in the minor groove (Figure 1A). Conformation B was more specific than Conformation A. In Conformation B, Gln 50 formed a water-mediated contact with Gua 5 and Thy 6* (TAATG_5_A_6_), and Asn 51 contacted Ade 2 in addition to Ade 3. The N-terminal arm was also more ordered in conformation B, with Lys 2 hydrogen bonded with the bases Ade 3 and Thy 2* in the minor groove.

### Crystal simulation

The flexibility of the interactions in the two conformations was investigated by MD of the crystallographic unit cell. During the simulation the four copies of model A and model B in the unit cell (Figure S1) showed some variation in mobility and conformation, probably due to fluctuating differences in their local instant environment. This agrees with the crystallographic information where the same fluctuations are likely responsible for the high B-factors.

**Figure 2:**
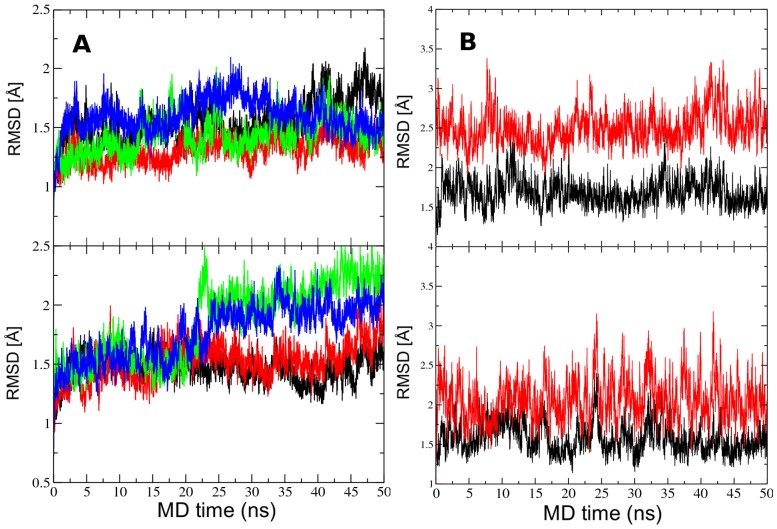
Agreement of simulations with the crystal structure. A) Stability of the crystal simulation. Mass weighted RMSD relative to the crystal structure (PDB 2H1K) (www.rcsb.org) for the eight Pdx1/DNA complexes comprising the unit cell of the 2H1K crystal during unrestrained molecular dynamics in the crystal environment. Conformation A is shown in the top panel and conformation B in the bottom panel. Different colors correspond to the different asymmetric units as specified in Figure S1. B) Stability of the solution simulation. Mass weighted root mean square deviation of the Pdx1/DNA complex computed with respect to the crystal Conformation A (black) and crystal conformation B (red) starting from Conformation A (top panel) and starting from Conformation B (bottom panel). Both simulations were closer to the crystal Conformation A. The two overhanging DNA bases in the crystal structure were excluded from the simulation.

The experimental molecular geometries were well preserved during the simulation (Figure 2A). The instantaneous mass-weighted root-mean-square deviations (RMSDs) with respect to the crystal structure were 2.5 Å or less. The average structure over all times and over the four replicas of each molecule was computed and its RMSD with respect to each of the crystal conformations was calculated. Note that the RMSD of the *average structure* is not the same as the average of the instantaneous RMSDs. (The average structure is of course a better approximation of the experimental structure than the various instantaneous structures). The RMSD of the *average structures* with respect to the original 2H1K coordinates were 0.86 Å and 1.10 Å for conformations A and B, respectively. We interpret this as validation of the model, the force field and the simulation protocol.

### Trajectories of the Pdx1/DNA complex in the crystal

In general the differences between conformations A and B were less pronounced after the crystal simulation. Arg 5 is the one residue to hydrogen bond consistently with the same bases in all 8 models in the unit cell, to Thy 1 and Gua −1* through the minor groove, as it does in the crystal structures. The major groove contacts are more variable. The hydrogen bond by Asn 51 with Ade 3 (CTA_2_ A_3_T) is lost consistently in Conformation A, while it is more stable in Conformation B (Figure S2A,B). Both Asn 51 and Gln 50 contact the phosphate backbone of the DNA in the major groove of Conformation A, with Ade 2 and Cyt 7*, respectively (Figure S2C). Only the Gln 50- Cyt 7* contact is accessible to Conformation B. These backbone contacts are characteristic of the partially specific Conformation A after the solution simulation.

In the crystal structure five phosphate contacts are unique to Conformation A by residues from helix 2 and 3: Arg 31, Lys 46, Gln 50, Arg 53 and Lys 57 (Figure 1B) [51]. During the simulation all of these contacts are also formed in conformation B except Arg 31 and Lys 46 with the phosphate backbone of Ade 8* (Figure S2D,E). Arg 31-Ade 8* is unique to Conformation A in solution too.

**Figure 3:**
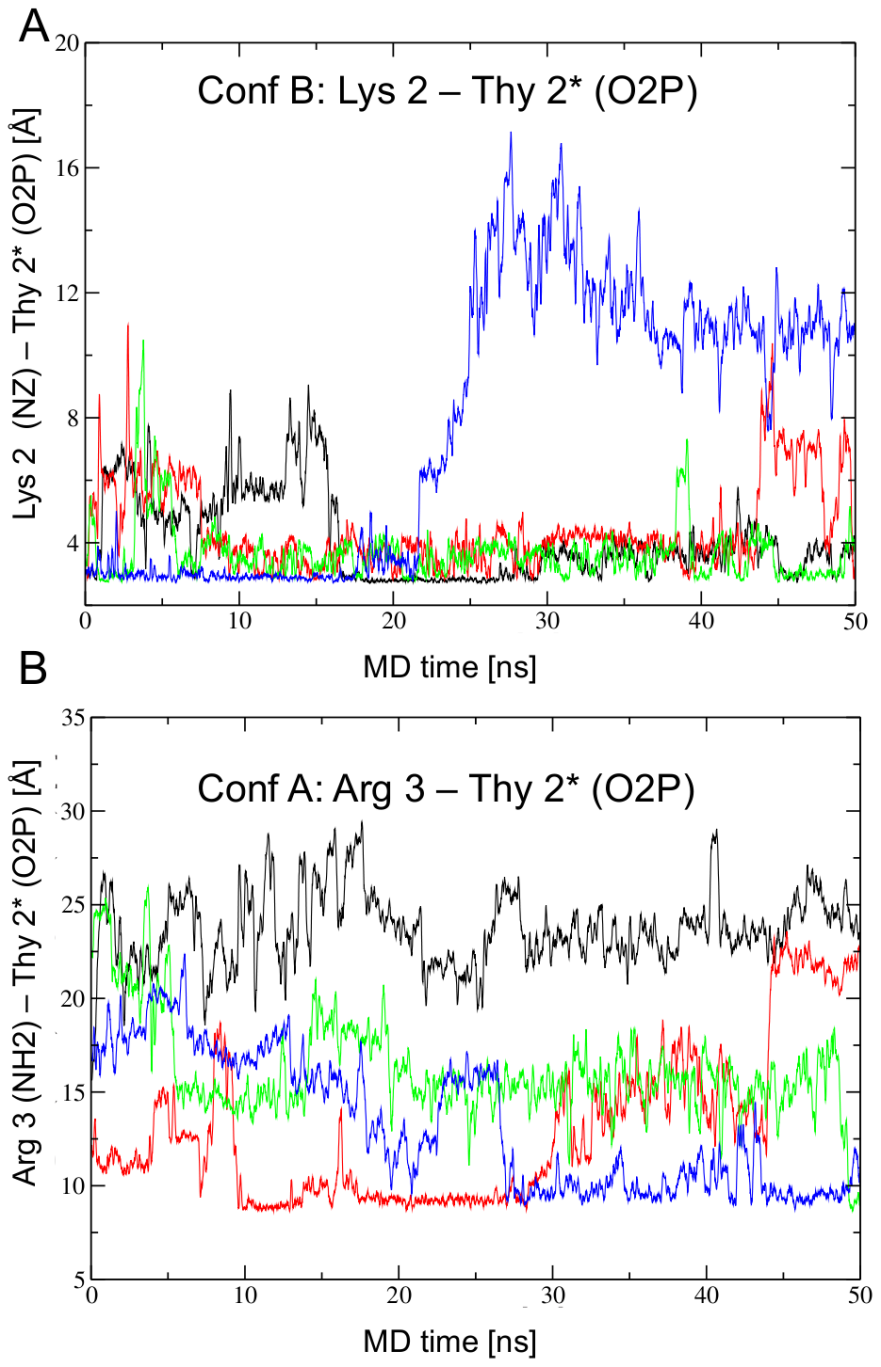
N-terminal arm contacts in the crystal simulation. A) In Conformation B, the N-terminal arm is mostly ordered with Lys 2 hydrogen bonding with the base of Thy 2* O2P. In model B4 (blue) the N-terminal arm escapes from the minor groove. B) The N-terminal arm in Conformation A starts the simulation disordered. Lys 2 never enters the minor groove, but Arg 3 enters the minor groove in model A2 (red), A4 (blue), and it seems to do so at the end of the simulation in A3 (green). The colors represent one of the four asymmetric units as depicted in Figure S1: A1, B1 black; A2, B2 red; A3, B3 green; A4, B4 blue.

While Arg 5 is consistently ordered in all homeodomain/DNA complexes, the residues N-terminal of Arg 5 are often disordered [7], [70], [71], [72]. In the crystal structure, these residues are ordered in Conformation B and disordered in Conformation A. During the simulation Lys 2 remains predominantly in the minor groove in Conformation B, hydrogen bonded with Thy 2* O2P (Figure 3A) but not Ade 3. In model B4 residues 1–4 of the N-terminal arm escape from the minor groove after about 20 ns and remain mobile. In Conformation A Lys 2 never enters the minor groove during the simulation. Interestingly, Arg 3 does enter the minor groove to contact Thy 2* for about 20 ns in model A2, and in model A4 (at 30–50 ns) and at the end of the simulation in model A3. (Figure 3B). Mobility of the N-terminal arm appears to be required for Arg 3 to enter the minor groove since the arm executes large motions in models A2 and A4, while these motions are restricted in models A1 and A3 by phosphate backbone contacts by Lys 2 or the acetylated N-terminus.

From the crystal structure we proposed that ordering of Lys 2 in the minor groove is stabilized by a network of contacts between Arg 43 and His 44 from helix 3 in the major groove and Arg 3 in the minor groove (Figure 1A) [51]. These interactions were maintained in conformation B during the crystal simulation: both Arg 3 and Arg 43 hydrogen bond with the phosphate backbone, with Thy 4 and Ade 3, respectively (Figure S3 A,B). The proximity of the guanidinium groups of Arg 3 and Arg 43 suggest pi-pi stacking. His 44 stabilizes the conformation of Arg 43 (Figure S3C). In model B4, after the N-terminal arm escapes the minor groove, the Arg 3-Thy 4 O2P and Arg 43-His 44 contacts are broken, consistent with their role in stabilizing the N-terminal arm in the minor groove.

In Conformation A the N-terminal 3 residues and the side chain of Arg 43 are disordered in the crystal structure. Arg 43 never associates stably with His 44 (Figure S3D) or Ade3 O2P, but forms a stable hydrogen bond (∼60% of the time) with Thy 4 O2P in model A2 and A3 (Figure S3E). In model A2, the Arg 43-Thy 4 O2P backbone contact correlates with insertion of Arg 3 in the minor groove to contact the base of Thy 2* (Figure 3B).

In summary, the simulation reduces somewhat the differences between Conformations A and B found in the crystal structure, particularly in the major groove. Three phosphate contacts are specific to Conformation A: by Asn 51 with Ade 2, and by Arg 31 and Lys 46 with Ade 8*. The contact by Arg 43 from the major groove with the phosphate backbone correlates with stabilizing the N-terminal arm. In Conformation B the N-terminal arm is mostly ordered with Lys 2 binding in the minor groove. In Conformation A the N-terminal arm is mostly disordered. Arg 3 enters the minor groove in models A2 and A4, suggesting a second position for the N-terminal arm not present in the crystal structure. We attributed the different contacts between the two conformations in the crystal structure to differences in DNA bending due to crystal packing [51]. The DNA bending of the crystal structure is maintained during the crystal simulation.

### Pdx1/DNA complex in aqueous solution

Simulations of the Pdx1/DNA complex in aqueous solution were initiated from both conformations reported in the 2H1K PDB (www.rcsb.org) [56] structure, and trajectories recorded for 50 ns. Mass-weighted RMSDs were calculated relative to the crystal Conformation A or the crystal Conformation B (Figure 2B). After an initial relaxation time, the simulations of both Conformation A and B resembled Conformation A more than B, indicating that Conformation A in the crystal structure, with the less bent DNA, is closer to the solution conformation. The average of the *instantaneous* RMSD values for complexes A and B relative to the experimental structure in Conformation A were 1.69 Å and 1.58 Å (black lines in top and bottom panels of Figure 2B), respectively; and relative to Conformation B were 2.51 Å and 2.08 Å, respectively (red lines in top and bottom panels Figure 2B).

### Specific DNA contacts in the major groove by conformation B

**Figure 4:**
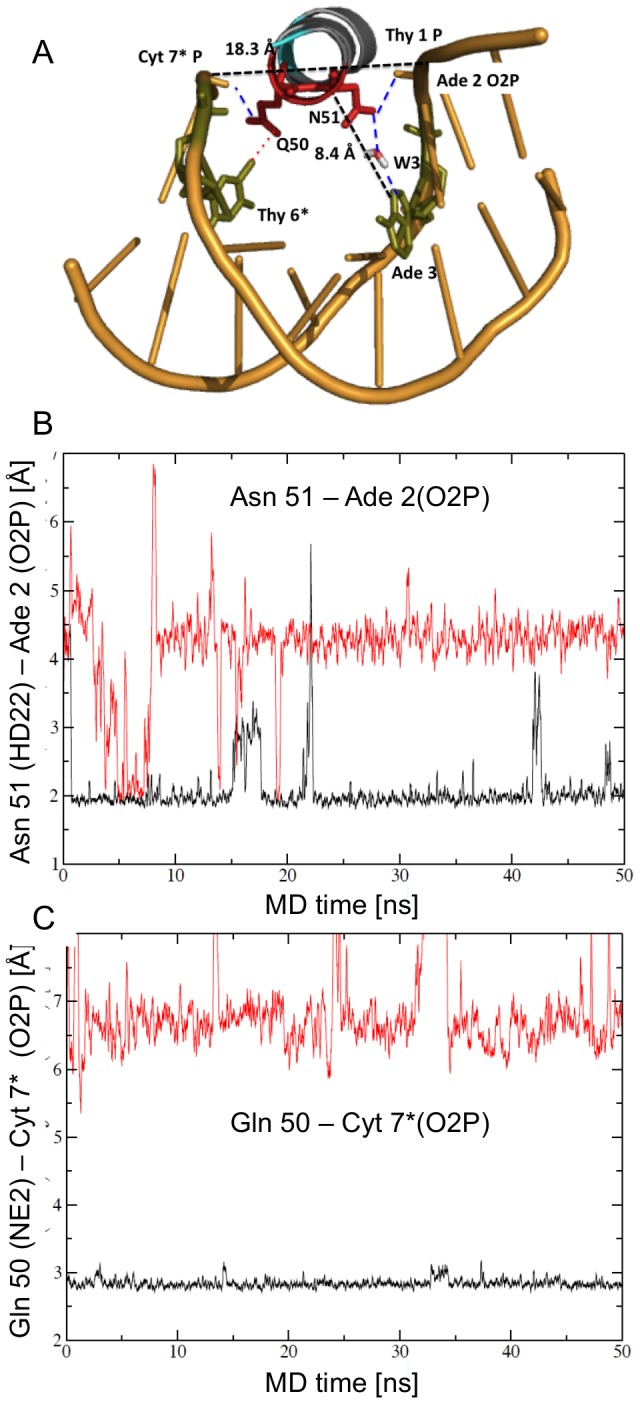
Contacts by helix 3 in the major groove of Conformation A during the solution simulation. A) Ribbon diagram looking into helix 3 and the major groove. Gln 50 and Asn 51 (labeled as Q50 and N51) contact the phosphate backbone only, with Cyt 7* and Ade 2, respectively (blue dashed lines). Gln 50 is within van der Waals contact of Thy 6* C7 (green, connect by a red dotted line). About 7% of the time a water molecule (W3) mediates contact between Asn 51 and Ade 3 (green). The position of helix 3 in the major groove is measured by the distance between Asn 51 C-alpha to Ade 3 N7 (8.4 Å), and the width of the major groove: Thy 1 P – Cyt 7* P (18.3 Å). The structure represents interactions at 30 ns of the simulation. B) Asn 51 contacts the backbone of Ade 2 O2P only in Conformation A (black), not in Conformation B (red). C) Gln 50 contacts the phosphate backbone of Cyt 7* O2P only in Conformation A (black), not in Conformation B (red).

**Figure 5:**
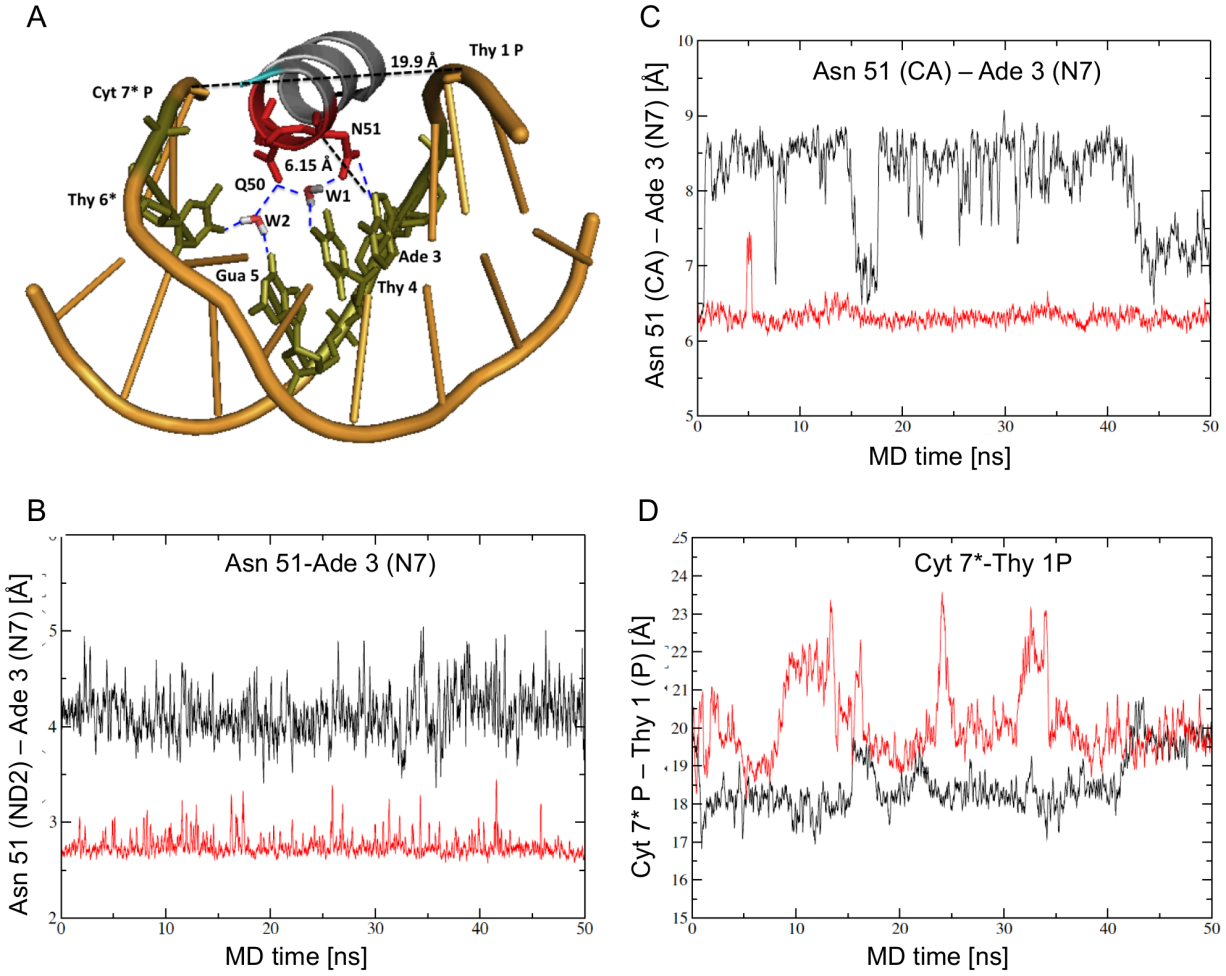
Contacts by helix 3 in the major groove of Conformation B during the solution simulation. A) Ribbon diagram looking into helix 3 and the major groove. Asn 51 contacts Ade 3 directly (blue dotted line). Gln 50 makes no direct contact with the DNA. About 20% of the time, water mediated contacts bridge between Gln 50 and Asn 51 (W1) and Gln 50 and DNA bases (in green) (W2). Distances show helix 3 binds farther in the major groove (Asn 51 C-alpha to Ade 3 N7 distance 6.15 Å), and the major groove is slightly wider than in Conformation A (Thy 1 P – Cyt 7* P distance 19.9 Å). B) Asn 51 forms a direct hydrogen bond with the base Ade 3 N7 only in Conformation B (red), not in Conformation A (black). C) and D) The position of the homeodomain differs in Conformation A and Conformation B during the solution simulation. C) Helix 3 binds closer to the DNA in Conformation B (red) than Conformation A (black), as measured by the distance between Asn 51 and Ade 3. D) The major groove is wider in Conformation B than Conformation A during most of the solution simulation, as measured by the Cyt 7* P-Thy 1 P distance. This is consistent with Pdx1 binding deeper in the DNA major groove in Conformation B.

The flexibility of the DNA during the simulation of both conformations resulted in an average straight helical axis with large fluctuations in the bending angle (not shown). We were therefore surprised that differences between the conformations persisted throughout the simulation. In conformation A, both Gln 50 and Asn 51 stably contacted the phosphate backbone in the major groove, with Cyt 7* and Ade 2, respectively (Figure 4). In contrast Asn 51 in conformation B did not contact the phosphate backbone of Ade 2 (Figure 4B) but formed a direct hydrogen bond with Ade 3 N7 (Figure 5 A,B). Periodically Asn 51 OD1 formed a second specific hydrogen bond with Ade 3 N6 (not shown). Gln 50 was too far from the DNA for a direct contact with the DNA bases, but during the simulation two water molecules sometimes (∼20% of the time) bridged between Gln 50 and Asn 51 and the bases of Thy 4, Gua 5 and Thy 6*. Water 1 (W1 in Figure 5A) also bridged between Gln 50 and Asn 51. These direct DNA contacts indicate that helix 3 continues to form more specific major groove contacts in Conformation B than in A.

### Ordering of the N-terminal arm

**Figure 6:**
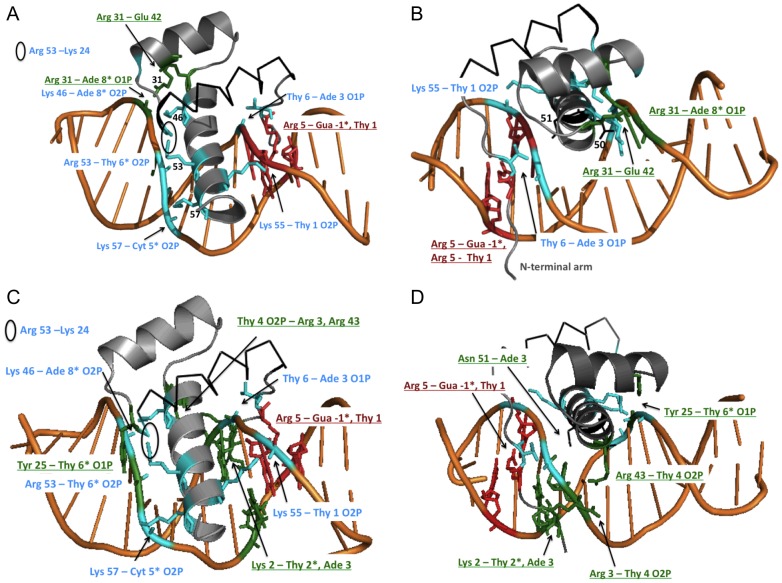
Interactions specific to Conformation A and B after 30 ns of the solution simulation. A) and B) Conformation A. C) and D) Conformation B. The base contacts by Arg 5 are identical in both conformations (red, underlined). Invariant (in Conformation A and B) contacts with the phosphate backbone include (cyan): Lys 46 – Ade 8* O2P, Arg 53 – Thy 6* O2P, Lys 57 – Cyt 5* O2P, Lys 55 – Thy 1 O2P, and Thy6 – Ade 3 O1P. The intramolecular hydrogen bond Arg 53 – Lys 24 is also conserved (circled). A) Hydrogen bond interactions by Pdx1 Conformation A (grey ribbon) with the DNA. Contacts unique to Conformation A (green, underlined) include a phosphate contact by Arg 31 with Ade 8*, in the major groove opposite the N-terminal arm, and the intramolecular contact between Arg 31 and Glu 42. B) Conformation A viewed looking into the minor groove. Residues 1–4 of the N-terminal arm are highly mobile in the solution simulation. Asn 51 and Glu 50 are shown in black lines. C) Hydrogen bonds by Pdx1 Conformation B (grey) with the DNA during the solution simulation, facing helix 3 in the major groove. Contacts unique to Conformation B (green, underlined) include a phosphate contacts by Tyr 25. D) Conformation B viewed looking into the minor groove. Arg 3 and Arg 43 hydrogen bond with the backbone of Thy 4 (green, underlined), assisting in stabilizing the N-terminal arm in the minor groove and the interaction by Lys 2 with the bases of Thy 2* and Ade 3.

The contacts by Arg 5 with Gua −1* and Thy 1 through the minor groove are conserved in the trajectories of both conformations (Figure 6). This remains the only direct hydrogen bond with a DNA base in Conformation A. The N-terminal residues 1–3 are initially ordered in conformation B, and Lys 2 continues to form a hydrogen bond with Thy 2* O2 for 35 ns of the simulation (Figure S4A). After that the N-terminus of Pdx1 moves outside of the minor groove, but Arg 3 and Arg 43 remain in contact with the DNA phosphate backbone, stabilizing the N-terminal arm (Figure S4 B–D). It is therefore plausible that Lys 2 would return to the minor groove in a longer simulation. In contrast to the crystal simulation, Arg 43 contacts Thy 4 O2P instead of Ade 3 O2P when the N-terminal arm is ordered in Conformation B (Figure S4 B,E, Figure S3A). His 44 does not contact Arg 43 during the solution simulation, unlike in the crystal simulation (Figure S3C).

In conformation A, Lys 2 begins the simulation outside of the minor groove and does not enter during the simulation. As seen in the crystal simulation, Arg 3 in Conformation A enters the minor groove towards the end of the simulation, but it never settles in a single position, contacting the base of Ade 3 only transiently. The residues that stabilize the N-terminal arm, Arg 3 and Arg 43, are more mobile in conformation A than conformation B (Figure S4 B–D). Arg 43 contacts Ade 3 only about a third of the trajectory (Figure S4E).

### Specific phosphate contacts

All five hydrogen bonds that were specific to Conformation A in the crystal structure were accessible to Conformation B. The Arg 31-Ade 3* contact is favored in Conformation A. The position of Arg 31 is stabilized through a hydrogen bond with Glu 42 in Conformation A (Figure S4F, Figure 6A,B). The contact between Tyr 25 and Thy 6* phosphate is restricted to Conformation B in the solution simulation (Figure S4G, Figure 6C,D). This contact was accessible to both conformations in the crystal structure.

### Position of the homeodomain with respect to the DNA

Clearly different contacts persist between conformations A and B through the 50 ns of the solution simulation. As mentioned, the DNA is highly flexible during the simulation indicating DNA bending cannot explain the conformational differences. Instead the overall positioning of the homeodomain of Pdx1 relative to the DNA differs for the two conformations, as indicated by the distance between Asn 51 CA and Ade 3 N7 (Conformation A : 8.0±0.7 Å; conformation B: 6.4±0.4 Å) (Figure 5C) and the width of the major groove, measured as the distance between the phosphate of Cyt 7* and Thy 1 (Conf A 18.6±0.8 Å, Conf B 20.1±1.0 Å, defined from the atom centers without subtracting 5.8 Å for the phosphorous van der Waals radius) (Figure 5D). Helix 3 is bound deeper in the major groove in conformation B allowing Asn 51 to contact Ade 3 N7 directly, which may account for the wider major groove (Figure 4A, 5A).

The difference in the positioning of the homeodomain between the conformations was not apparent in the crystal structure: the distance between Asn 51 CA and Ade 3 N7 was about 6.2 Å for both conformations. The major groove width (Cyt 7* P – Thy 1 P) was different: 19.4 Å in Conformation A and 20.8 Å in Conformation B. During the crystal structure simulation, the distance between helix 3 and Ade 3 varies between 6 and 8 Å in conformation A, and between 6 and 7 Å in conformation B. The constraints of the crystal packing therefore prevented repositioning of the homeodomain.

## Discussion

### The MD simulation

MD has been applied to DNA/homeodomain complexes previously to study protein-DNA and water mediated contacts [73], the role of salt bridges [74], the role of residue 50 [75], [76], folding properties of the N-terminal arm [77], and other studies [67], [78], [79], [80], [81]. In general these simulations are initiated from a unique structure, assuming that the simulation will explore the relevant conformational space. In the current study we applied MD to investigate two distinct DNA binding conformations of the Pdx1 homeodomain to determine if the differences were the result of crystal packing. Simulations were carried out in the context of a crystal unit cell and in solution. The solution simulations generated two different conformations of the Pdx1/DNA complex depending on the initial conformation derived from the crystal structure. Both conformations were stable during the 50 ns simulation. The current study demonstrates the real possibility of multiple stable conformations that are not accessible during limited simulation times.

The AMBER force field ff99SB [57], [58] used in the simulations reported in this work is considered state-of-the-art, and includes several refinements for DNA simulations [59], [82], [83], [84]. Present computer capabilities allow fully atomistic simulations, minimizing artifacts. The DNA and protein in these simulations are fully solvated with explicit waters; in a relatively large box under periodic boundary conditions (as opposed to spherical water clusters that may experience various surface potential discontinuities at the cluster-vacuum or cluster-continuum interface [85], [86]); with a correct treatment of electrostatics [87], [88]. Crystal simulations at constant pressure and temperature (NPT) that reproduce the crystallographic cell and symmetries have traditionally been used to test and tune force fields, as they allow direct comparison with experiments, and will also reproduce packing effects [52], [53], [54], [55], [89], [90], [91], [92].

### Two stable conformations of Pdx1 bound to DNA

After the solution simulation Conformation B bound DNA specifically while Conformation A bound with limited specificity. The unique interactions in the two conformations were due to different positions of the homeodomain in the major groove of the DNA, with helix 3 buried deeper in the major groove in Conformation B than in Conformation A (Figure 4A and 5A). In Conformation B Asn 51 interacts directly with Ade 3 in the major groove, and Lys 2 of the N-terminal arm contacts bases through the minor groove. The proximity of helix 3 to the DNA in Conformation B facilitates ordering bridging water molecules between the protein and DNA, with Gln 50 and Asn 51 (Figure 5A). These bridging water molecules were not observed in the Pdx1 crystal structure, but were observed in the related Antennapedia structure [71].

What determines the position of helix 3 in the major groove? We previously attributed the presence of two Pdx1/DNA conformations in the crystal structure to the curvature of the DNA in conformation B [51]. The differences between the two conformations identified in the crystal structure diminished during the crystal simulation, despite maintaining the average curvature of the DNA in conformation B. In contrast, differences between the two conformations increased during the solution simulations. A comparison of the Antp homeodomain/DNA complex by NMR and crystallography indicated contacts by Arg 43 with Ade 3, and movements of Gln 50 and Asn 51 in the NMR structure that could not be explained from the crystal structure [71]. These contacts are consistent with the motions of the Pdx1 homeodomain in the solution simulation. Clearly the crystal lattice constrained the Pdx1/DNA conformation, suggesting caution when interpreting crystal structures of protein/DNA complexes.

What properties of the two conformations in the crystal structure directed the solution simulations toward the specific (starting from Conformation B) versus less specific (starting from Conformation A) complexes? The average DNA sequence was straight during the solution simulation of both conformations, indicating that DNA bending was not the primary cause. In the crystal structure helix 3 was oriented at slightly different angles relative to the DNA in the two conformations. The specific phosphate contacts formed by Conformation A in the crystal structure are accessible to Conformation B during the solution simulation. Already in the crystal structure the contacts are less specific in Conformation A. The configuration that defines Conformation A includes: Gln 50 contacting the phosphate backbone at base 7*, Asn 51 contacting Ade 3 but not Ade 2, and the disordered N-terminal residues (Figure 1B). The contacts that define Conformation B include: Asn 51 contacting Ade 2 and Ade 3 in the major grove, Gln 50 making a water mediated contact with DNA bases at positions 5 and 6, and the ordered N-terminal arm with Lys 2 contacting Thy 2* and Ade 3.

### The DNA “bound” state consists of multiple conformations

The MD simulations presented here suggest multiple conformations are possible for the N-terminal arm in the minor groove and for the helix-turn-helix domain in the major groove. In both conformations, Arg 5 contacts Gua −1* and Thy 1 through the minor grove (Figure 6). The most stable (longest-lived) configuration for the N-terminal arm of Pdx1 consists of Lys 2 inserted in the minor groove and Arg 3 outside of the minor grove contacting the phosphate backbone and Arg 43 (Figure S4A–D). In Conformation A, Arg 3 inserts in the minor groove and contacts the base Thy 2* for some time in the crystal simulation (Figure 3B). This configuration resembles the configuration in the Scr-Exd DNA complex (the *Drosophila* homolog of Hox5-Pbx1) with a 14 residue N-terminal extension of Scr, including the YPWM Pbx1 binding motif [25]. In that structure the N-terminal arm was ordered with Arg 3 inserted in the minor groove but contacting the phosphate backbone. The authors suggested that Arg 3 is positioned by a His residue along the N-terminal extension. Therefore while binding of the Pdx1 monomer may favor base contacts by Lys 2 in the minor groove, other protein interactions may favor Arg 3 positioned in the minor groove.

The MD simulation also distinguishes two orientations of the helix-turn-helix domain in the major groove. An alternate orientation of the recognition helix was previously characterized for the Mata2 homeodomain bound to a nonspecific DNA sequence [93]. In this structure the homeodomain was rotated with respect to the consensus binding site, altering interactions in the major groove and eliminating contacts by the N-terminal arm in the minor groove. A second paper noted that the Hox homeodomains in the HoxA9-Pbx1 and HoxB1-Pbx1 complexes were oriented differently in the major grove, altering base contacts [94], [95]. In contrast to these examples, *the two conformations of the Pdx1 homeodomain are bound to the same DNA sequences of the consensus-binding site*. In the less specific Conformation A of Pdx1, Arg 5 makes base-specific contacts through the minor groove, like the specific conformation. Many of the same phosphate contacts position helix 3 in the major groove, by Thy 6, Arg 31, Arg 53, Lys 55, and Lys 57. But helix 3 of Conformation A is too far from the DNA bases to form direct hydrogen bonds; instead Gln 50 and Asn 51 contact the phosphate backbone (Figure 4A).

One interpretation of the partially specific Conformation A is that it represents a DNA binding intermediate in search of the specific DNA binding conformation B. The Pdx1 homeodomain binds nonspecific DNA with just 20-fold lower affinity than the consensus site [23], [51]. Other homeodomains also bind DNA with low specificity, as noted for Mata2 and Antennapedia [96], [97]. The stability of the less-specific Conformation A during the MD simulation suggests it might be populated when the Pdx1 monomer binds nonspecific DNA sequences.

Pdx1 binds to thousands of DNA sites *in vivo*, as measured by ChIP-Seq, including sequences distinct from the consensus binding sequence [13], [98]. In binding a specific DNA sequence, both conformations A and B may be present as two of an ensemble of DNA-bound conformations. In this scenario the DNA sequence and other protein interactions stabilize a subset of this ensemble. The diversity of interactions might explain the myriad of functions accomplished by Pdx1.

### Coordination between the major and minor groove

In our previous paper we proposed that Arg 43 and Arg 3 bridge between the major and minor grooves to order the N-terminal arm in Conformation B, suggesting some synergistic interactions between the helical and N-terminal domains. Many studies conclude that the N-terminal arm contributes to DNA binding specificity of homeodomains [24], [45], [46], [47], [99], [100], [101]. Synergy between the major and minor groove has been noted for chimeric homeodomains, which generally require mutations in the N-terminal arm and the recognition helix to change specificity between homeodomain factors [24], [99]. In a survey of all *Drosophila* homeodomains, specificity determinants for DNA binding originate from both the recognition helix and N-terminal residues [20], [102].

Like other Hox factors, Pdx1 binds DNA cooperatively with PBC class homeodomains, such as Pbx1 [30]. Extensions of the N-terminal arm to the “YPWM” motif enhance DNA binding specificity of Hox factors, exposing “latent specificity” among the eight Hox paralogs through interactions with Pbx1 [5], [27], [103]. But minor groove contacts do not explain all of the sequence preferences observed. For example a comparison of two structures by the *Drosophila* Hox-Pbx1 heterodimer Scr-Exd bound to different DNA sequences demonstrated conformational changes in the extended N-terminal linker as well as contacts in the major groove [25]. In the context of Pbx1, the consensus-binding site for the Hox factors is generally not TAAT, necessitating different DNA interactions by the N-terminal arm and recognition helix. The MD simulations reported here suggest that the DNA and protein context may promote “specific binding” by restricting the ensemble of accessible conformations available to the homeodomain on the DNA.

Even though longer MD simulations are needed to probe “rare” conformational transitions and to completely characterize the relative stability of the different conformations, the fact that completely independent X-ray studies support the existence of these two conformations lends validity to our conclusions. These can be summarized as follows. Conformation A represents a partially specific DNA bound configuration with a single base contact by Arg 5 in the minor groove. Conformation B represents the specific Pdx1 conformation, forming additional direct and water-mediated contacts with DNA bases by Asn 51 and Gln 50 in the major groove, and by Lys 2 in the minor groove. These conformations differ in the position of helix 3 in the major groove and indicate some of the inherent flexibility of homeodomains in binding DNA. The stability of both conformations suggests they both play a role in the free energy landscape of the complex: either as stable minima or a kinetically trapped intermediate (Conformation A) in search of a global minimum (Conformation B). Flexibility in DNA binding of the homeodomain may be important in allowing Pdx1 to fulfill its multiple functional roles, particularly in binding non-consensus DNA sequences or in the presence of DNA binding partners. A source of diversity of homeodomain function may derive from distinct bound states with differing degrees of DNA binding specificity. Further structural and MD studies of Pdx1 to different DNA sequences and in the presence of partner proteins are necessary to characterize DNA binding in the context of authentic enhancers.

## Supporting Information

Figure S1 Packing of the Pdx1/DNA complex in the unit cell of the crystal structure. Each asymmetric unit contains two Pdx1 monomers in Conformation A (yellow) and Conformation B (magenta), and two DNA helices (colored black, red, green and blue in asymmetric unit 1, 2, 3 and 4, respectively). The packing constraints differ for each model during the crystal simulation. B1 and A4 are the most constrained by crystal contacts, including the N-terminal arm; in A1 and B4 the helices are constrained but not the N-terminal arm; and A2, A3, B2 and B3 are not constrained by crystal contacts.
(TIF)

Figure S2 Different contacts between Conformations A and B in the crystal simulation. A) In Conformation A the hydrogen bond between Asn 51 and Ade 3 is lost in all models. B) In Conformation B the contact between Asn 51 and Ade 3 is more consistent than Conformation A, but still lost in all but model B2. C) In Conformation A when Asn 51 is not contacting the base of Ade 3 it frequently forms a hydrogen bond with the phosphate backbone of Ade 2. This contact is favored in the solution simulation of Conformation A, and is not formed in Conformation B (see Figure 3). D) In Conformation A Lys 46 contacts the phosphate backbone of Ade 8*. This is one of the backbone-specific contacts in Conformation A. E) In Conformation B the side chain of Lys 46 is more mobile than in Conformation A.
(TIF)

Figure S3 Contacts stabilizing the N-terminal arm in Conformation B during the crystal simulation. The N-terminal arm is stabilized by contacts by Arg 43 in the major groove and Arg 3 in the minor grove. A) In Conformation B, Arg 43 contacts the phosphate backbone of Ade 3. This contact is broken in model B4 (blue) after the N-terminal arm escapes the minor groove. B) Arg 3 generally contacts the phosphate backbone of Thy 4, and C) Arg 43 contacts His 44 in Conformation B, but D) not in Conformation A. E) In Conformation A Arg 43 is generally mobile except in models A2 (red) and A3 (green) in which Arg 43 contacts the phosphate backbone of Thy 4. In model A2 (red) this contact correlates with insertion of Arg 3 into the minor groove, before 30 ns (Figure 2B).
(TIF)

Figure S4 Contacts stabilizing the N-terminal arm in Conformation B during the solution simulation. A) Lys 2 remains in the minor groove in Conformation B (red) for about 35 ns, contacting the base of Thy 2*. The N-terminal arm is disordered in conformation A (black). B) Arg 43 contacts the phosphate backbone of Thy 4 in Conformation B (red). C) Arg 3 contacts the phosphate backbone of Thy 4 in Conformation B for about 25% of the trajectory. D) Arg 43 and Arg 3 may interact through pi-pi stacking in Conformation B only. E) In Conformation A, Arg 43 contacts the phosphate backbone of Ade 3 during about 1/3 of the solution simulation (black). F) In Conformation A Glu 42 interacts with Arg 31 (black) and stabilizes the phosphate contact between Arg 31 and Ade 8*, the only specific phosphate contact remaining in Conformation A. G) A hydrogen bond between Tyr 25 OH and Thy 6* O1P is unique to Conformation B (red).
(TIF)
