# Supplementary figures and images for: Binding Polymorphism in the DNA Bound State of the Pdx1 Homeodomain
Source: PLoS Comput Biol. 2013 Aug 8;9(8):e1003160. doi: 10.1371/journal.pcbi.1003160 (PMC3738460; doi:10.1371/journal.pcbi.1003160)

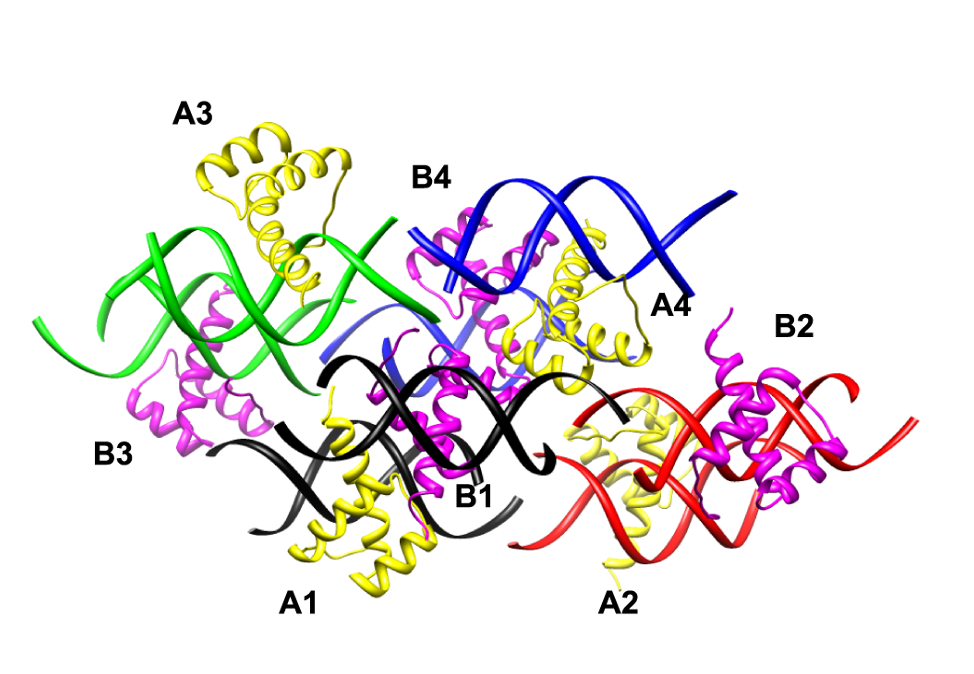

Supplement: Figure S1 — Packing of the Pdx1/DNA complex in the unit cell of the crystal structure. Each asymmetric unit contains two Pdx1 monomers in Conformation A (yellow) and Conformation B (magenta), and two DNA helices (colored black, red, green and blue in asymmetric unit 1, 2, 3 and 4, respectively). The packing constraints differ for each model during the crystal simulation. B1 and A4 are the most constrained by crystal contacts, including the N-terminal arm; in A1 and B4 the helices are constrained but not the N-terminal arm; and A2, A3, B2 and B3 are not constrained by crystal contacts. (TIF) [file pcbi.1003160.s001.tif]

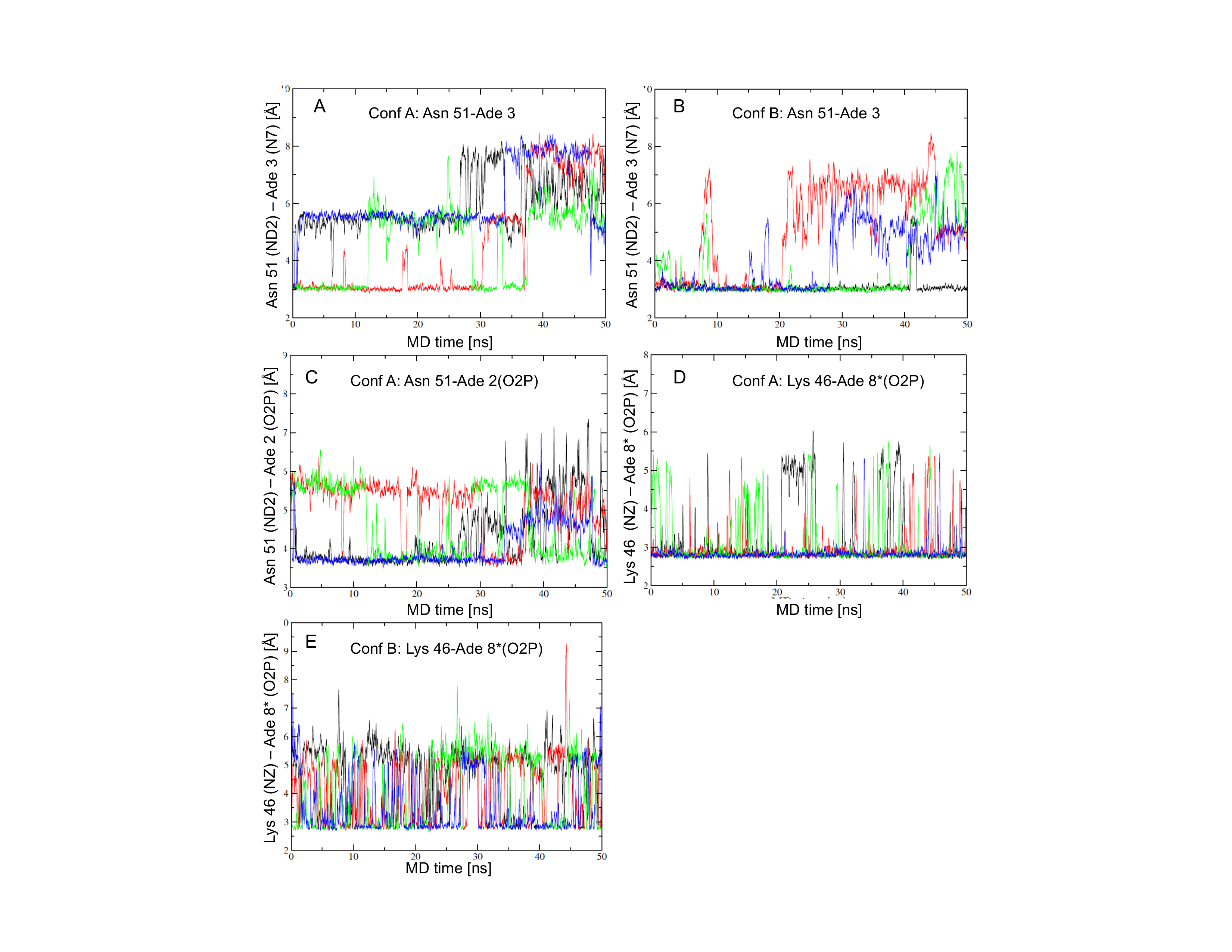

Supplement: Figure S2 — Different contacts between Conformations A and B in the crystal simulation. A) In Conformation A the hydrogen bond between Asn 51 and Ade 3 is lost in all models. B) In Conformation B the contact between Asn 51 and Ade 3 is more consistent than Conformation A, but still lost in all but model B2. C) In Conformation A when Asn 51 is not contacting the base of Ade 3 it frequently forms a hydrogen bond with the phosphate backbone of Ade 2. This contact is favored in the solution simulation of Conformation A, and is not formed in Conformation B (see Figure 3). D) In Conformation A Lys 46 contacts the phosphate backbone of Ade 8*. This is one of the backbone-specific contacts in Conformation A. E) In Conformation B the side chain of Lys 46 is more mobile than in Conformation A. (TIF) [file pcbi.1003160.s002.tif]

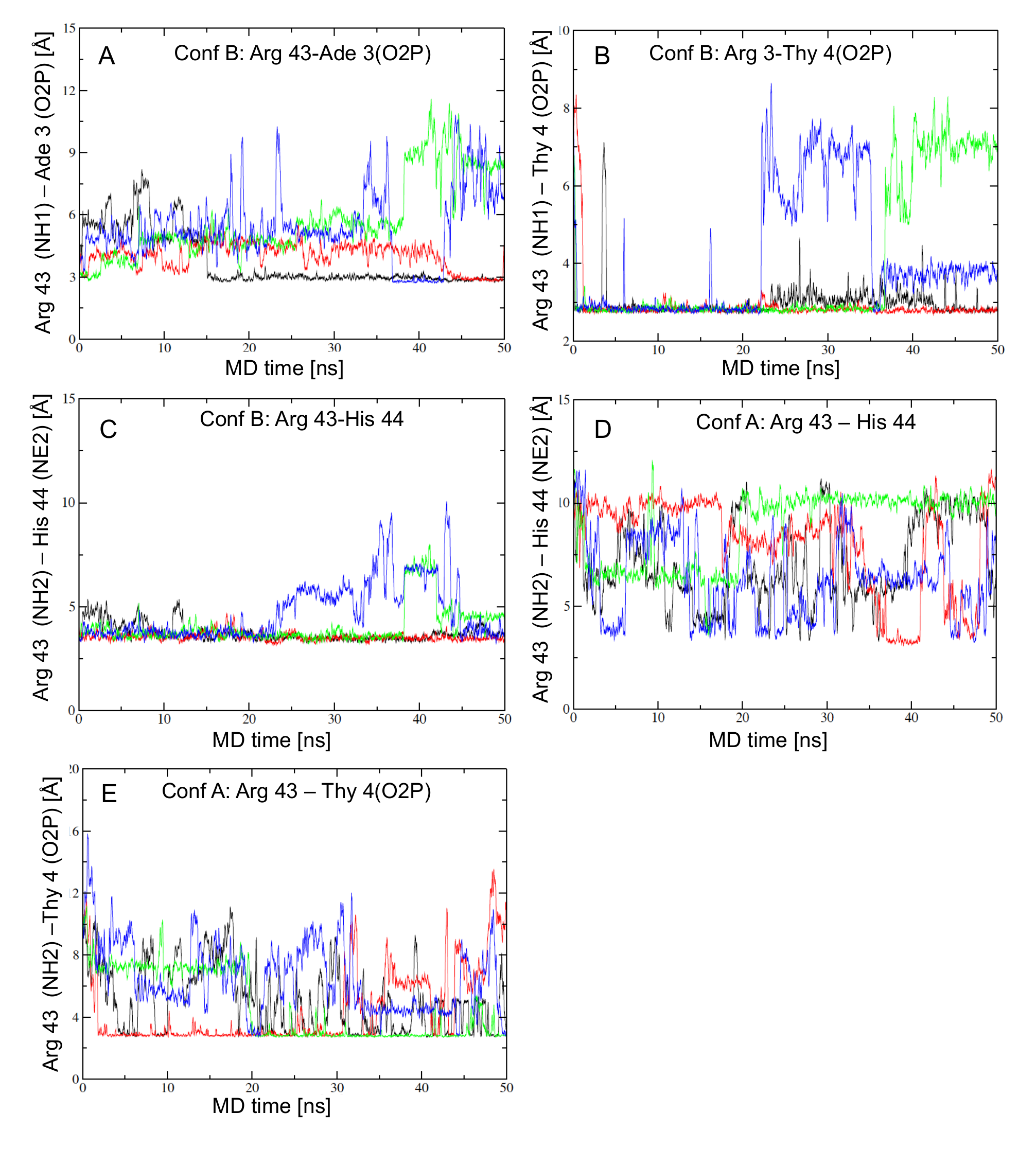

Supplement: Figure S3 — Contacts stabilizing the N-terminal arm in Conformation B during the crystal simulation. The N-terminal arm is stabilized by contacts by Arg 43 in the major groove and Arg 3 in the minor grove. A) In Conformation B, Arg 43 contacts the phosphate backbone of Ade 3. This contact is broken in model B4 (blue) after the N-terminal arm escapes the minor groove. B) Arg 3 generally contacts the phosphate backbone of Thy 4, and C) Arg 43 contacts His 44 in Conformation B, but D) not in Conformation A. E) In Conformation A Arg 43 is generally mobile except in models A2 (red) and A3 (green) in which Arg 43 contacts the phosphate backbone of Thy 4. In model A2 (red) this contact correlates with insertion of Arg 3 into the minor groove, before 30 ns (Figure 2B). (TIF) [file pcbi.1003160.s003.tif]

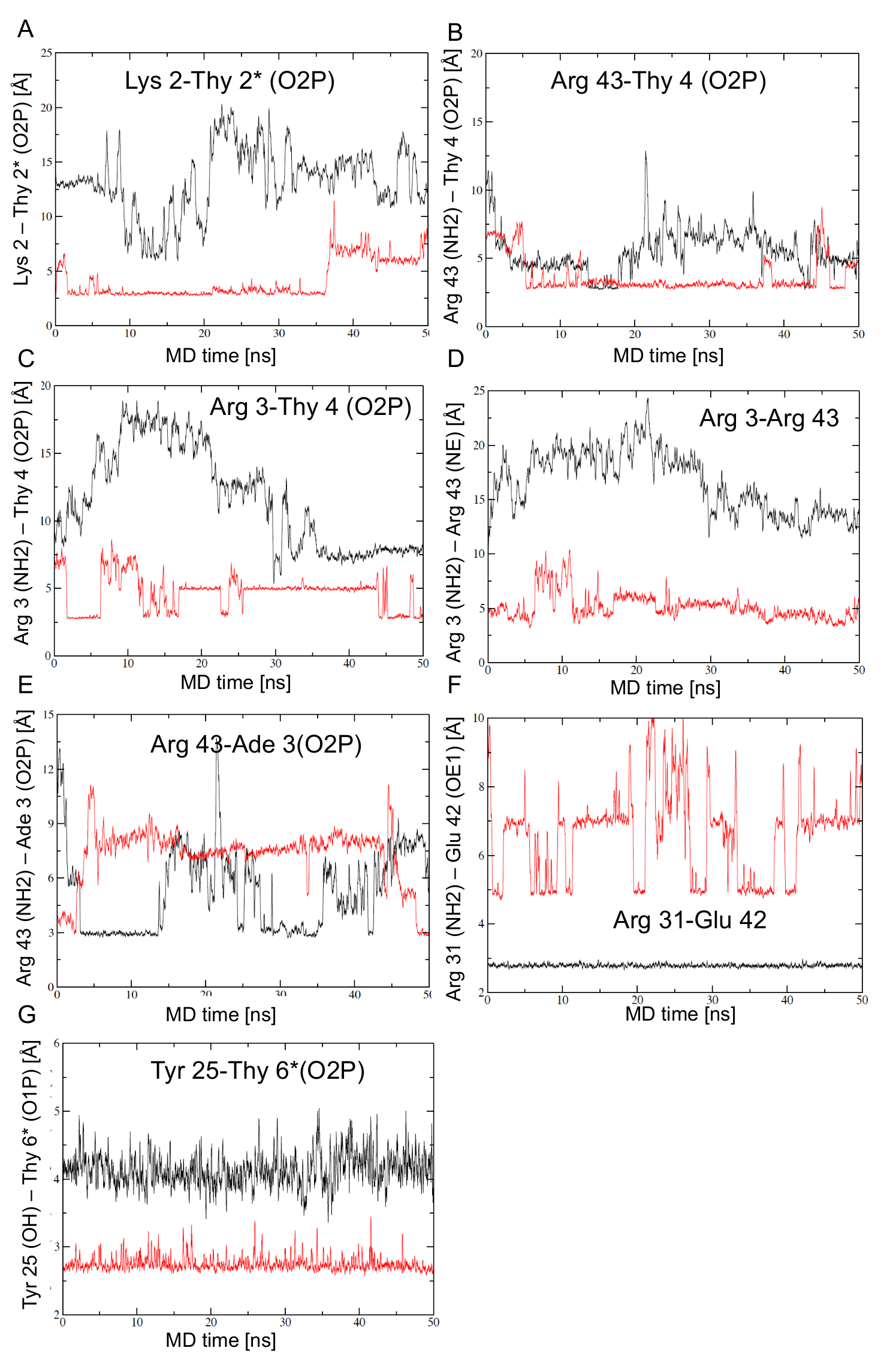

Supplement: Figure S4 — Contacts stabilizing the N-terminal arm in Conformation B during the solution simulation. A) Lys 2 remains in the minor groove in Conformation B (red) for about 35 ns, contacting the base of Thy 2*. The N-terminal arm is disordered in conformation A (black). B) Arg 43 contacts the phosphate backbone of Thy 4 in Conformation B (red). C) Arg 3 contacts the phosphate backbone of Thy 4 in Conformation B for about 25% of the trajectory. D) Arg 43 and Arg 3 may interact through pi-pi stacking in Conformation B only. E) In Conformation A, Arg 43 contacts the phosphate backbone of Ade 3 during about 1/3 of the solution simulation (black). F) In Conformation A Glu 42 interacts with Arg 31 (black) and stabilizes the phosphate contact between Arg 31 and Ade 8*, the only specific phosphate contact remaining in Conformation A. G) A hydrogen bond between Tyr 25 OH and Thy 6* O1P is unique to Conformation B (red). (TIF) [file pcbi.1003160.s004.tif]
